# Italian style coffee consumption and metabolically dysfunctional-associated steatotic liver disease (MASLD): a cohort population study in Southern Italy

**DOI:** 10.3389/fnut.2026.1797230

**Published:** 2026-03-19

**Authors:** Manuela Siani, Caterina Bonfiglio, Leonilde Bonfrate, Rossella Donghia, Maria Noemy Pastore

**Affiliations:** 1 Unit of Data Science, National Institute of Gastroenterology-IRCCS “Saverio de Bellis”, Bari, Italy; 2 Center of Nutrition for the Research and the Care of Obesity and Metabolic Diseases, National Institute of Gastroenterology IRCCS “Saverio de Bellis”, Bari, Italy; 3 University San Raffaele, Rome, Italy

**Keywords:** coffee, cohort study, Italian style coffee, liver disease, MASLD

## Abstract

Metabolic dysfunction-associated steatotic liver disease (MASLD) is currently the most prevalent chronic liver disease globally, and its incidence is rising consistently along with the prevalence of type 2 diabetes mellitus and overweight/obesity. Coffee has a unique chemical composition consisting of numerous compounds that contribute to metabolic and liver health. Nevertheless, it is not yet clear what dose of coffee should be safely consumed to positively impact MASLD. The current study aimed to examine the association between the consumption of Italian-style coffee in terms of cups per day, and the protection against MASLD within a cohort from southern Italy. The results indicated that the risk of developing MASLD decreased as daily coffee consumption increased when compared to consuming less than one cup per day (1 cup: 0.479 95% CI 0.244; 0.944; 2 cups: 0.468 95% CI 0.242; 0.905; 3 cups: 0.449 95% CI 0.225; 0.893), with the greatest benefit at 4–6 cups (OR: 0.407; 95% CI 0.186; 0.893). These findings suggest that moderate coffee consumption, specifically Italian-style coffee, may offer potential benefits due to its unique chemical composition and preparation method. This type of coffee may provide specific protective effects on liver and metabolic health. Given its cultural significance and positive metabolic attributes, Italian-style coffee has the potential to be recognized as a functional food for the prevention of MASLD.

## Graphical Abstract

**Figure:**
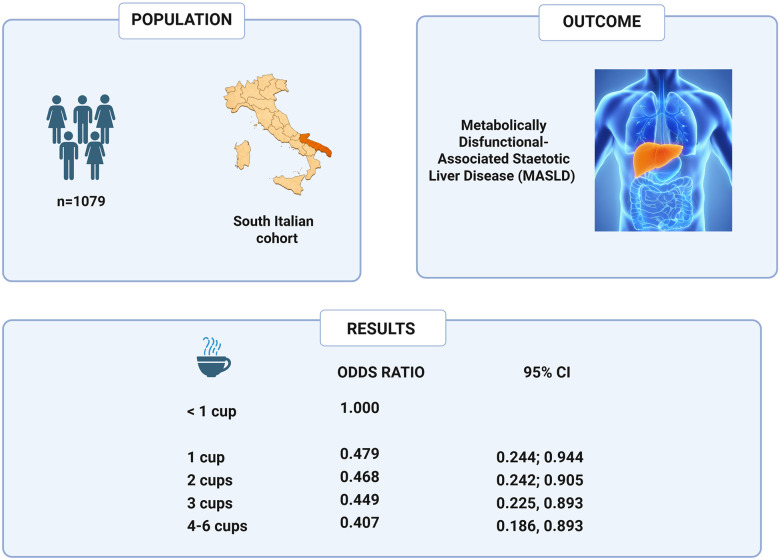
Graphical abstract (Created in BioRender: https://BioRender.com/3zg2zhd).

## 1 Introduction

Metabolic dysfunction-associated steatotic liver disease (MASLD) is defined as steatotic liver disease (SLD) characterized by the presence of one or more cardiometabolic risk factors and the consumption of alcohol at non-harmful levels (1). This updated definition removes the stigma associated with non-alcoholic fatty liver disease (NAFLD) (2, 3). Although the two definitions are separate, numerous cohort studies have demonstrated a high degree of overlap in NAFLD-related evidence in individuals with MASLD (4).

MASLD is currently the most prevalent chronic liver disease globally, with a prevalence of over 30% (5) and incidence rates continuing to rise, particularly in males and overweight/obese individuals (6). This condition causes a significant social and economic burden and impact that requires prompt management in public health and politics (7).

The pathogenic mechanism of MASLD is based on excessive triglyceride (TG) synthesis in the liver, caused by an imbalance between fatty acid (FA) uptake and oxidation. Fatty acids can come from diet and adipose tissue, but they can also be synthesized *de novo* from non-lipidic precursors. *De novo* lipidic lipogenesis (DNL) appears to be the primary contributor to TG accumulation in MASLD patients (8). Insulin resistance, the key pathological component of obesity and type 2 diabetes mellitus (T2DM), is a major factor in the progression of MASLD, as it causes an increase in hepatic *de novo* lipogenesis, promoting triglyceride synthesis (9, 10). The accumulation of free fatty acids (FFAs) in the liver causes mitochondrial damage, resulting in inflammation and oxidative stress, which act as amplifiers of MASLD. Additionally, the accumulation of FFAs leads to lipotoxicity in liver tissue, which contributes to further progression of the disease (11). The risk of progression to metabolic dysfunction–associated steatohepatitis (MASH) is approximately 10–30%, especially in the presence of T2DM (4). Further developments with a worse prognosis may include fibrosis, cirrhosis, and hepatocellular carcinoma related to MASH.

The management of MASLD requires a multidisciplinary approach to control the progression of the disease in both its hepatic and cardiometabolic components. A comprehensive lifestyle change, including weight loss, diet, and exercise, is the key to success in adults with MASLD (1). One of the dietary approaches that has garnered significant interest from the scientific community regarding MASLD is coffee. Coffee, one of the most popular beverages worldwide, offers intriguing possibilities, although its implications are not yet fully understood (12).

Coffee has a unique chemical composition consisting of over 100 active compounds that contribute to its numerous beneficial effects on human health (13). Particularly, its functional compounds, especially chlorogenic acids (CGAs), caffeine, diterpenes, trigonelline, and melanoidins, perform numerous functions, some of which are still being studied (14).

Previous studies have shown a dose-response relationship between coffee consumption and a reduction in the risk of T2DM, chronic kidney disease mortality (CKD), and all-cause mortality (15, 16). However, the connection between coffee consumption and liver health is still not fully understood. Evidence indicates that drinking coffee has clear beneficial effects on the development of fibrosis in individuals with MASLD (17, 18). Despite this, the evidence supporting a link between coffee consumption and the occurrence of MASLD remains weak (15, 19).

According to some studies, including prospective cohort studies, regular unsweetened coffee drinkers appear to have a lower risk of developing MASLD/NAFLD, and the effect appears to be progressive as the number of cups of coffee consumed increases (20–22).

Nevertheless, some studies suggest that there is no association between coffee consumption and the onset of MASLD (23). A randomized clinical trial found that neither chlorogenic acid nor caffeine was more effective than a placebo in reducing hepatic fat, stiffness, and other hepatic outcomes in patients with diabetes and NAFLD. This finding may be attributed to the fact that the doses of CGAs and caffeine used in the trial corresponded to approximately two cups of coffee (24).

It is important to note that most studies investigating the effects of coffee on MASLD do not specify the roasting techniques or brewing methods used. These factors can significantly influence the levels of caffeine and active compounds in coffee, leading to varying health effects (15, 20). Unfiltered brewing methods, such as espresso, moka, French press, or Turkish coffee, appear to be much more effective for liver health outcomes compared to filtered coffee, as they contain higher concentrations of diterpenes and CGAs (20).

This observational study seeks to further investigate the relationship between coffee consumption, particularly in terms of dosage, and the occurrence of MASLD within a large cohort of the Italian population. The study will also consider traditional coffee brewing methods, such as espresso and moka.

## 2 Materials and methods

### 2.1 Study population

The NUTRIHEP study is a cohort initiated in 2005–2006, using a systematic random sample of attendees over 18 years from the Putignano Primary Care Physicians' list procedure (25, 26). From 2015 to 2018, all participants in the Nutrihep cohort were invited to the first follow-up. A total of 1,426 participants responded and adhered to the same protocol used during the original enrolment. All provided informed consent after being informed about the medical data involved. For simplicity, the study was designed as a cross-sectional study, focusing only on follow-up measurements. The study was approved by the Ethical Committee of the Minister of Health (DDG-CE-792/2014, on 14 February 2014).

### 2.2 Data collection

During follow-up visits, participants completed all assessments specified in the protocol. Trained physicians and/or nutritionists conducted interviews to gather data on sociodemographic details, health status, personal history, and lifestyle factors. This encompassed tobacco use history, dietary intake, educational background, occupational profile, and marital status.

Participants' weight and height were measured while wearing only underclothing and no shoes. We used an electronic balance (SECA©) to record weight to the nearest 1 kg, and a wall-mounted stadiometer (SECA©) to measure height to the nearest 1 cm. Blood pressure (BP) was measured following international guidelines (27, 28) and the average of three measurements was calculated. Participants completed the European Prospective Investigation into Cancer and Nutrition (EPIC) Food Frequency Questionnaire (FFQ) independently to gather information about their eating habits (29, 30). Nutritionists validated all responses and uploaded the questionnaire into a custom online tool. Afterwards, the entered nutritional data was transformed into micro- and macro-nutrients.

The following blood measurements were taken: fasting serum glucose (FSG), fasting insulin, HbA1c, triglycerides, total cholesterol, LDL-C, HDL-C, AST, ALT, ALP, GGT, ferritin, and high-sensitivity C-reactive protein. Analyses were performed using the COBAS 8000 autoanalyser (ROCHE Diagnostics SPA, Monza, Italy). Insulin resistance was estimated using the Homeostasis Model Assessment of Insulin Resistance (HOMA-IR), calculated by the following formula (31).


$$\begin{array}{l} \text{HOMA}-\text{IR}=\text{FSG }\left(\text{mg}/\text{dL}\right)\times \text{fasting Insulin }\left(\text{μIU}/\text{mL}\right)/405. \end{array}$$

All subjects had standardized ultrasound exams using a Hitachi H21 Vision (Hitachi Medical Corporation, Tokyo, Japan). The liver parenchyma was examined with a 3.5 MHz transducer. A scoring system was used to assess hepatic fat content semi-quantitatively.

The degree of hepatic fat infiltration was determined based on liver echotexture, hepatic echo penetration, hepatic blood vessel clarity, and hepatic diaphragm differentiation in echo amplitude (32). Supplementary Figure S1 illustrates the ultrasound scan board employed in the study to determine the steatosis grade.

### 2.3 Outcome assessment

MASLD is defined by the presence of hepatic steatosis combined with at least one of the following cardiometabolic risk factors: (1) BMI over 25 kg/m^2^ or waist circumference exceeding 94 cm in men and 80 cm in women; (2) fasting serum glucose of 100 mg/dL or higher, 2-h post-load glucose of 140 mg/dL or higher, HbA1c of 5.7% or higher, or being on specific medication; (3) blood pressure of 130/85 mmHg or higher, or on specific medication; (4) plasma triglycerides at or above 150 mg/dL, or on specific medication; (5) plasma HDL cholesterol below 40 mg/dL in men and below 50 mg/dL in women, or on specific medication. Additionally, the MASLD definition continues to restrict alcohol intake (similar to NAFLD) in those with steatosis to an average daily intake of 20–50 g for women and 30–60 g for men (1, 33, 34).

**Figure 1:**
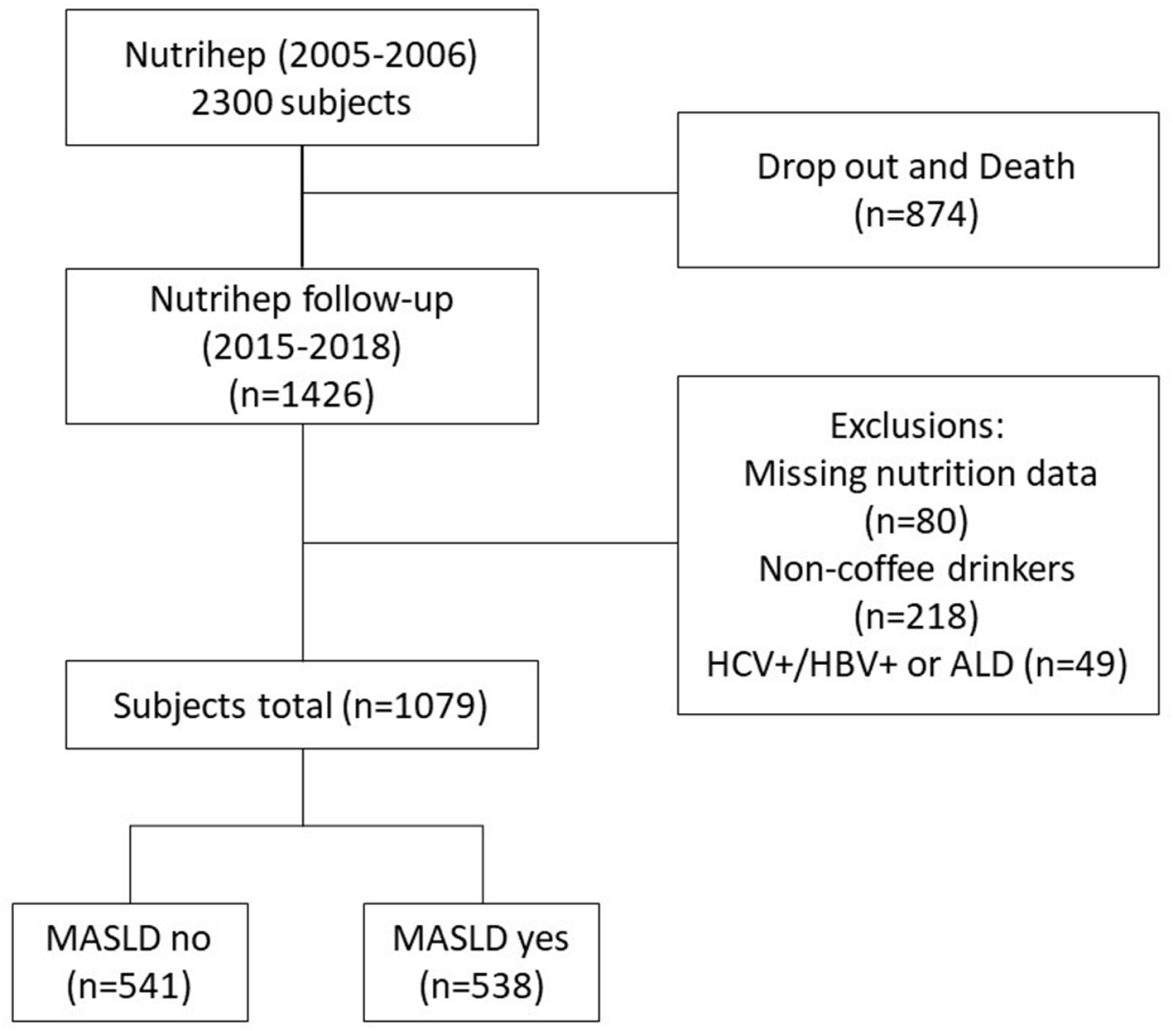
Flow chart.

Finally, other forms of liver disease coexisting with MASLD, such as MASLD + HCV, HBV, were ruled out to avoid altering the natural history of the disease (Figure 1) (1).

### 2.4 Exposure variable

The main exposure in our analysis was coffee consumption recorded through self-reported information in the EPIC semi-quantitative food frequency questionnaire (FFQ). Each participant was asked whether they had drunk coffee in the previous year and how many cups they had drunk per day. From the FFQ, we included “espresso” and “moka” preparations; no other coffee preparations were included in the section. As for the quantity, we used a median value between espresso and moka, following the latest Dietary Reference Values of Nutrients and energy for the Italian population (35), resulting in 40 ml. The number of cups of coffee was then classified as < 1, 1, 2, 3, 4–6.

### 2.5 Confounding variables

Covariates were selected based on previous research and both clinical and statistical judgment regarding their potential link to MASLD. After evaluating collinearity risks, we included demographic and lifestyle variables such as age, sex (Male vs. Female), daily energy intake, coffee with milk, beyond the cups consumed daily (No vs. Yes), sugar in coffee (No vs. Yes), smoking status (never vs. current), rMED, education level, superalcoholic intake (ml/day), as well as anthropometric and clinical parameters like BMI and diastolic blood pressure, alongside laboratory measures including HDL, AST/ALT, and glycemic categories (normoglycemia, prediabetes, diabetes).

### 2.6 Statistical analysis

Subject characteristics were presented as means and standard deviations (Mean ± SD) or medians and interquartile ranges for continuous variables, and as frequencies and percentages (%) for categorical variables. We used the Wilcoxon test or *t*-test to compare continuous variables between two groups, based on the distribution of the variables. For categorical variables, the χ2 test was used to assess differences.

In addition, logistic regression models were developed to estimate odds ratios (OR) and 95% confidence intervals (CI), with MASLD as the outcome and coffee consumption (measured in cups per day) as both a continuous and categorical exposure variable (< 1, 1, 2, 3, 4–6 cups per day).

Model a was univariate, while model b was adjusted for age, sex (Female vs. Male), BMI, Diastolic Blood Pressure, Daily kilocalorie, Sugar in coffee (No vs. Yes), coffee with milk, beyond the cups consumed daily (No vs. Yes), Smoke (Never vs. Current), rMED, Education, Superalcoholic (ml/day), HDL, AST/ALT, and glycemic categories (normoglycemia < 100 mg/dL, prediabetes 100–125 mg/dL, diabetes ≥ 126 mg/dL), and the estimated coefficients were transformed into Odds Ratio (OR).

Initially, confounding variables were chosen based on existing literature. Then, the minimum absolute reduction and selection (LASSO) method was used to reduce the number of candidate predictors and highlight the most valuable ones for building the model (36).

The Variance Inflation Factor (VIF) was assessed to detect multicollinearity, and confounders with VIF > 5 were removed (37). The two-tailed probability level was set at 0.05 to test the null hypothesis of non-association.

The analyses were conducted with StataCorp 2025 Stata Statistical Software: Release 19 (College Station, TX, USA: StataCorp LLC.).

## 3 Results

### 3.1 Participants characteristics

Table 1 displays the main characteristics of the 1,079 subjects in the Nutrihep cohort who reported drinking coffee, categorized by the presence or absence of MASLD. The sample included 538 subjects with MASLD (49.86%). Of the 591 women, 45.9% had MASLD, and among the 488 men, 267 (54.7%) had MASLD.

**Table 1:** Characteristics of participants by MASLD Nutrihep Study, Putignano (BA), Italy 2015–2018.

**Variables**	**Whole sample^c^**	**MASLD**		***p*-value^d^**
		**No**	**Yes**	
*N* (%)	1,079	541 (50.14)	538 (49.86)	
**Exposure variable**				
**Coffee cups (day) (%)**				
<1	77 (7.1)	35 (45.45)	42 (54.55)	0.38
1	285 (26.4)	142 (49.82)	143 (50.18)	
2	362 (33.5)	172 (47.51)	190 (52.49)	
3	232 (21.5)	128 (55.17)	104 (44.83)	
4–6	123 (11.4)	64 (52.03)	59 (47.97)	
**Demographic characteristics**				
**Gender (%)**				
Female	591 (54.77%)	320 (54.15)	271 (45.85)	0.004
Male	488 (45.23%)	221 (45.29)	267 (54.71)	
Age (yrs)^a^	54.56 (14.02)	49.43 (13.57)	59.72 (12.50)	<0.001
**Anthropometric and clinical parameters**				
SBP (mmHg)^b^	120 (110–130)	115.00 (100.00–120.00)	130.00 (120.00–140.00)	<0.001
DBP (mmHg)^b^	80 (70–80)	80.00 (70.00–80.00)	80.00 (80.00–80.00)	<0.001
BMI (kg/m^2^)^b^	27.11 (24.24–30.85)	25.02 (22.63–27.34)	29.94 (26.78–33.33)	<0.001
Weight (kg)^b^	73.68 (14.77)	67.25 (12.10)	80.15 (14.38)	<0.001
Waist (cm)^b^	91.00 (82.00–100.00)	83.00 (76.00–90.00)	99.00 (91.00–106.00)	<0.001
**Hypertension (%)**				
No	701 (68.32%)	417 (59.49)	284 (40.51)	<0.001
Yes	325 (31.68%)	98 (30.15)	227 (69.85)	
**Dyslipidemia (%)**				
No	866 (84.49%)	454 (52.42)	412 (47.58)	0.001
Yes	159 (15.51%)	61 (38.36)	98 (61.64)	
**Diabetes (%)**				
No	955 (93.08%)	503 (52.67)	452 (47.33)	<0.001
Yes	71 (6.92%)	12 (16.90)	59 (83.10)	
**Blood test**				
HbA1c (mmol/mol)^a^	38.12 (7.01)	36.58 (5.08)	39.67 (8.24)	<0.001
HOMA^b^	1.44 (1.02–2.24)	1.16 (0.78–1.54)	1.87 (1.27–3.01)	<0.001
ALT (U/L)^b^	20.00 (16.00–25.00)	18.00 (15.00–23.00)	22.00 (17.00–28.00)	<0.001
ɤGT (U/L)^b^	14.00 (11.00–20.00)	13.00 (10.00–17.00)	16.00 (12.00–23.00)	<0.001
AST (U/L)^b^	21.00 (18.00–24.00)	20.00 (17.00–23.00)	22.00 (19.00–25.00)	<0.001
TG (mg/dL)^b^	79.00 (54.00–126.00)	68.00 (46.50–98.00)	101.00 (67.00–153.50)	<0.001
C-reactive protein (mg/dL)^b^	0.11 (0.09–0.25)	0.09 (0.09–0.18)	0.16 (0.09–0.33)	<0.001
TC (mg/dL)^b^	190.00 (167.00–215.00)	187.00 (165.00–210.00)	192.50 (169.00–218.00)	0.018
HDL (mg/dL)^b^	49.00 (41.00–58.00)	52.00 (44.00–61.00)	47.00 (39.00–55.00)	<0.001
Glucose (mg/dL)^a^	95.81 (17.65)	90.61 (10.83)	101.05 (21.28)	<0.001
**Glycemic categories (%)**				
< 100 mg/dL	946 (75.02)	572 (60.47)	347 (39.53)	<0.001
100–125 mg/dL	249 (19.75)	76 (30.52)	173 (69.48)	
≥126 mg/dL	66 (5.23)	7 (10.61)	59 (89.39)	
ALP (U/L)^a^	52.96 (15.95)	50.08 (15.49)	55.87 (15.90)	<0.001

^a^As means and Standard Deviations. ^b^As median and Interquartile Range.^c^Percentages calculated for the column. Otherwise, percentages are calculated for the row. ^d^Wilcoxon rank-sum tests or *t*-test for continuous variables to compare two groups based on the distribution of variables, and the χ2 test for categorical variables. MASLD, Metabolic dysfunction-associated steatotic liver disease; rMED, Relative Mediterranean Diet; BMI, Body Mass Index; SBP, Systolic Blood Pressure; DBP, Diastolic Blood Pressure; HbA1c, Glycosylated Hemoglobin; HOMA, Homeostasis Model Assessment; ALT, Alanine Amino transferase; ɤGT, Gamma Glutamyl Transferase; AST, Aspartate Amino transferase; TG, Triglycerides; TC, Total Cholesterol; HDL-C, High-Density Lipoprotein Cholesterol; ALP, Alkaline Phosphatase Level.

As shown in Table 1, participants with MASLD were generally older [59.72 years (±12.50)] and predominantly female. They had a higher prevalence of hypertension and hyperlipidaemia, along with higher BMI and weight, with means of 30.41 (4.78) and 80.15 (14.38), respectively.

The median number of cups consumed per day among the subjects in our sample is 2. The difference in coffee consumption distribution between those with MASLD and those without is not statistically significant; therefore, the two outcome categories are approximately equal.

As shown in Table 1, 63% (363 of 569) of participants with lower education levels had MASLD, compared with 31.6% (43 of 136) among university graduates with higher education levels. Blood parameters were higher in patients with MASLD, with statistically significant differences between the two groups (see Supplementary Table S1).

### 3.2 The associations between coffee daily intake and the occurrence of MASLD

Table 2 shows the results of logistic regression models analysing the relationship between MASLD and coffee consumption, presented as either a continuous or categorical variable.

**Table 2:** Logistic regression analysis of the association between daily coffee intake and MASLD.

	**Model a**			**Model b**		
**Daily consumption**	**OR** ^a^	***p*** **-value**	**95% CI**	**OR** ^a^	***p*** **-value**	**95% CI**
<1 cup	1.000			1.000		
1 cup	0.839	0.496	0.506; 1.391	0.479	0.033	0.244; 0.944
2 cups	0.920	0.742	0.562; 1.508	0.468	0.024	0.242; 0.905
3 cups	0.677	0.140	0.403; 1.136	0.449	0.023	0.225; 0.893
4–6 cups	0.768	0.366	0.434; 1.360	0.407	0.025	0.186; 0.893
Daily consumption (cups)	0.928	0.178	0.833; 1.034	0.843	0.027	0.725; 0.980

Daily coffee consumption was expressed both as a continuous variable and in cups.

^a^No MASLD reference category. The data are reported as Odds Ratios (ORs) and 95% C.I. Model a univariate; Model b adjusting for age, sex (Female vs. Male), BMI, Diastolic Blood Pressure, Daily Energy, Sugar in coffee (No vs. Yes), Milk and coffee (No vs. Yes); Education, Smoke (never vs. current), rMED, Superalcoholic (ml/day), HDL-C, AST/ALT, and Glycemic categories (normoglycemia < 100 mg/dL, prediabetes 100–125 mg/dL, diabetes ≥126 mg/dL). rMED, Relative Mediterranean Diet; HDL-C, High-Density Lipoprotein; AST, Aspartate Amino Transferase; ALT, Alanine Amino Transferase; HOMA, Homeostasis Model Assessment.

The results of the logistic regression model reported in Table 2 indicate a reduction in the risk of developing MASLD as the number of cups of coffee consumed per day increases (1 cup: 0.479 95% CI 0.244; 0.944; 2 cups: 0.468 95% CI 0.242; 0.905; 3 cups: 0.449 95% CI 0.225; 0.893 and 4–6 cups: 0.407 95% CI 0.186; 0.893) compared to consuming less than one cup per day. Supplementary Table S3 presents the results of the logistic regression analysis, including all correction and adjustment variables in the multivariate model, together with estimates of their effects.

Specifically, compared to individuals drinking less than one cup daily:

Drinking 1 cup per day reduced the risk by approximately 52.1 % [Odds Ratio (OR): 0.479];

Drinking 2 cups per day reduced the risk by approximately 53.2 % (OR: 0.468);

Drinking 3 cups per day reduced the risk by approximately 55.1 % (OR: 0.449);

Drinking 4–6 cups per day showed the significant reduction approximately 59.3% (OR: 0.407) (see Table 2).

These findings suggest a potential protective association of coffee consumption against MASLD, based on a model adjusted for age, sex (Female vs. Male), BMI, diastolic blood pressure, daily kilocalorie, sugar in coffee, milk and coffee (No vs. Yes); smoke (Never vs. Current), rMED, education, superalcoholic (ml/day), HDL, AST/ALT, and glycemic categories.

The odds ratio (OR) of 0.843 (95% CI: 0.725; 0.980) is just below 1, suggesting a slight negative association between daily coffee consumption and the risk of developing MASLD.

## 4 Discussion

This study investigates the relationship between coffee consumption, measured by the number of cups consumed per day, and the outcome of MASLD in a cohort of 1,079 coffee drinkers from the Nutrihep study. Drinking coffee represents a fundamental ritual in Italian culture, and approximately 93% of consumers prefer espresso, prepared either with an espresso machine or a Moka pot, both of which are characterized by a pressure-based extraction method (38). Other popular Italian variants, such as caffè macchiato and cappuccino, include the addition of milk; for this reason, data in this study were adjusted for milk addition. Italian coffee is typically served in small cups, with an average standardized serving of 30 ml, allowing for accurate comparisons of intake across participants.

This study demonstrates a significant inverse association between Italian-style coffee consumption and the risk of developing metabolic dysfunction–associated steatotic liver disease (MASLD). The results revealed a dose–response relationship: as daily coffee consumption increased, the risk of MASLD steadily decreased. Even consuming just 1 cup of coffee each day positively influenced MASLD risk reduction, with the most significant benefits observed in those who consumed a moderate amount, approximately 4 to 6 cups per day. These findings suggest that Italian-style coffee, due to its distinctive chemical composition and preparation method, may confer specific protective effects on liver and metabolic health.

**Figure 2:**
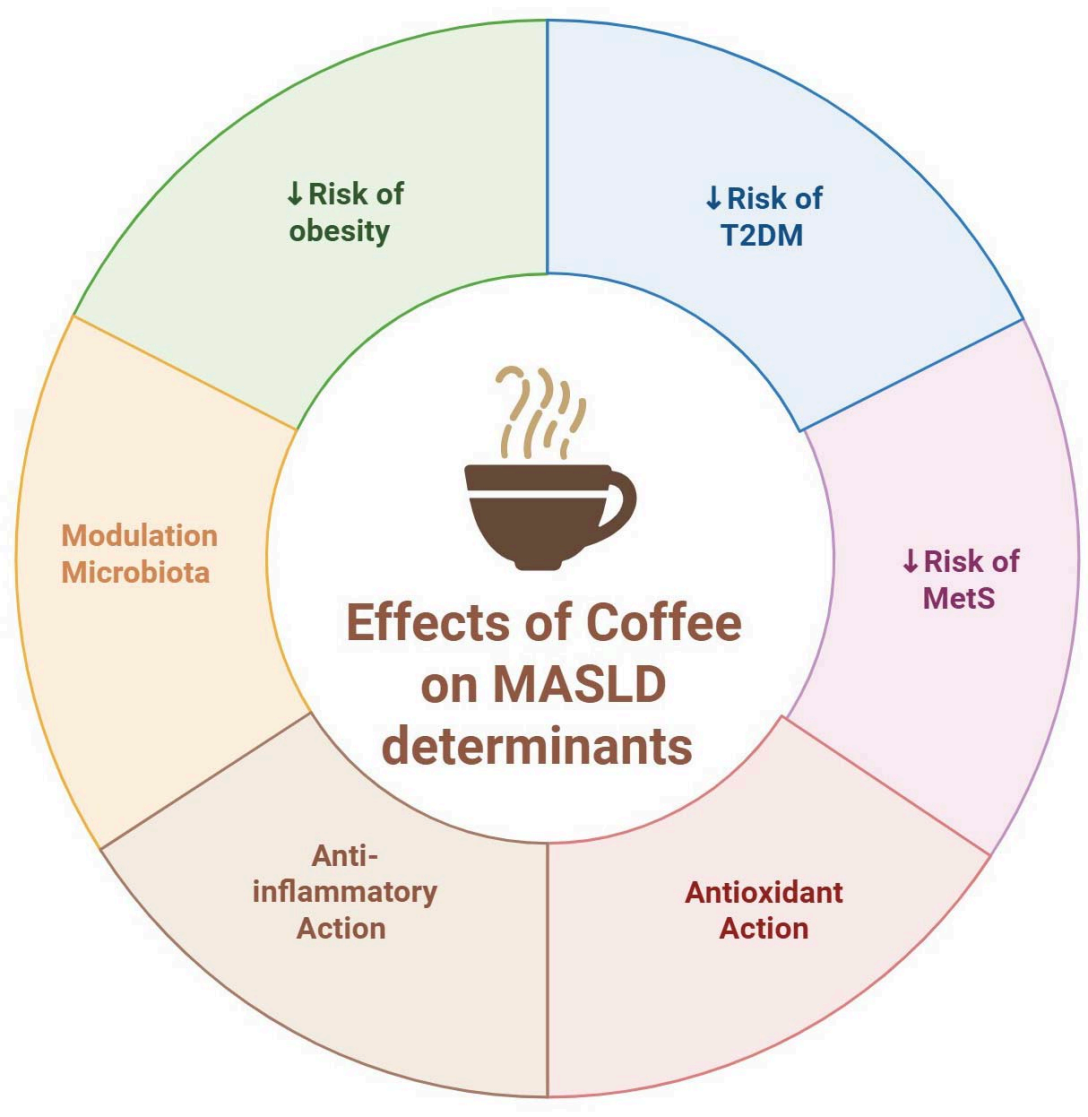
Effects of coffee on MASLD determinants (Created in BioRender: https://BioRender.com/g5hl66t).

The beneficial effects of coffee can be understood in the context of the multifactorial pathophysiology of MASLD. The disease is closely associated with metabolic abnormalities such as obesity, visceral adiposity, insulin resistance, dyslipidemia, and arterial hypertension. Previous literature supports the protective influence of coffee on many of these determinants (12). Several studies have documented an inverse relationship between coffee intake and the prevalence of obesity, metabolic syndrome, and T2DM (39). For instance, research from the Spanish SUN cohort demonstrated that moderate coffee intake is associated with a reduced incidence of metabolic syndrome, further supporting coffee's potential benefits in metabolic health management (40). The primary impacts of coffee on the determinants of MASLD are illustrated in Figure 2.

Coffee is a complex beverage containing numerous bioactive compounds, including alkaloids, phenolic acids, flavonoids, terpenoids, diterpenes, and melanoidins (14). The final chemical composition is strongly influenced by factors such as the type of bean (Arabica vs. Robusta), roasting degree, and brewing method (20). Italian-style preparations, namely espresso and moka, are characterized by high concentrations of diterpenes and CGAs, which may account for their unique biological effects compared to other brewing techniques (15, 20).

**Figure 3:**
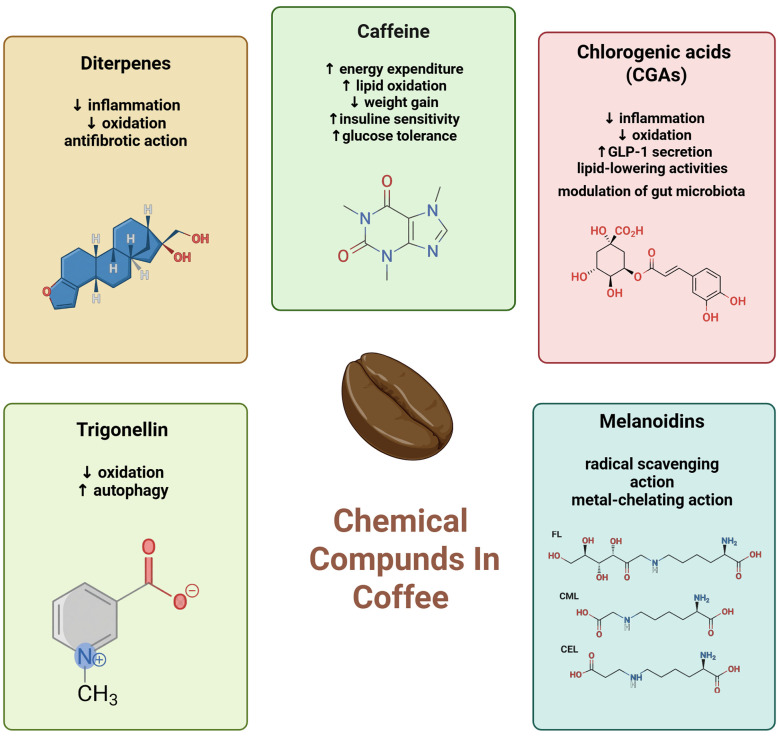
Chemical compounds in coffee and their effect on MASLD determinants (Created in BioRender: https://BioRender.com/r9epcm3).

Each chemical compound present in coffee influences the determinants of MASLD, acting on several pathways as summarized in Figure 3.

Caffeine, the main alkaloid in coffee, is present at higher levels than in most other foods and beverages (41), and has been associated with increased energy expenditure, enhanced lipid oxidation, and protection against weight gain by modulating gastrointestinal hormone secretion, enhancing insulin sensitivity, and maintaining glucose tolerance (42). These mechanisms may explain the well-established inverse relationship between coffee intake and T2DM risk, which follows a dose-dependent pattern, with the strongest protection observed in high consumers (≥ 6 cups/day) (15). Additionally, caffeine has been reported to inhibit the connective tissue growth factor (CTGF), a trigger factor that activates the stellate cells with consequent increased deposition of extracellular matrix proteins present in the fibrotic tissue (43, 44). Chlorogenic acids (CGAs), the predominant phenolic acids in coffee, exhibit antioxidant, anti-inflammatory, and lipid-lowering activities (45). Experimental evidence suggests that CGA attenuates hepatic steatosis and inflammation in high-fat diet models, likely via modulation of gut microbiota and stimulation of GLP-1 secretion (46, 47). Human studies further indicate that coffee rich in CGAs, such as light-roast preparations, may improve body composition and lipid metabolism (47). Diterpenes, particularly cafestol and kahweol, are abundant in Italian-style coffee. These compounds exert multiple hepatoprotective effects, including antioxidant, anti-inflammatory, antifibrotic, and anti-carcinogenic actions (48–50). Trigonelline, another major alkaloid, has been demonstrated to mitigate hepatic steatosis and oxidative stress in both cellular and animal models, potentially by restoring autophagy and reducing endoplasmic reticulum stress (51). Melanoidins, which are formed during the roasting process, provide antioxidant and anti-inflammatory effects through mechanisms such as radical scavenging and metal chelation (52, 53). However, the effectiveness of melanoidins is influenced by the degree of roasting; dark roasting increases melanoidin content but reduces heat-sensitive CGAs (20, 54).

The Italian brewing methods, especially espresso, allow higher retention of lipophilic compounds such as diterpenes and oils, as well as higher CGAs compared to filtered or instant preparations. Additionally, Arabica beans, commonly used in Italy, are richer in polyphenols and CGAs than Robusta, enhancing their antioxidant potential (20). These compositional characteristics may explain why the protective associations observed in this study are particularly evident for Italian-style coffee and not for other brewing techniques (20, 21, 55).

Emerging evidence indicates that coffee modulates the composition of gut microbiota by decreasing Bacteroides and Firmicutes while increasing Acinetobacter and Proteobacteria, promoting an anti-obesogenic profile (13). Given that dysbiosis contributes to MASLD pathogenesis (56), this microbiota-mediated mechanism may represent another pathway linking coffee to liver health.

Regarding blood pressure, the relationship appears to be complex. While acute caffeine intake can transiently elevate systolic and diastolic blood pressure in hypertensive individuals (57), chronic consumption does not appear to have hypertensive effects, and green coffee extract may even reduce blood pressure levels (57, 58). This suggests a net neutral or beneficial cardiovascular profile with habitual consumption.

Our results align with those from Mediterranean cohort studies showing reduced odds of metabolic syndrome with moderate coffee intake (40). Since MASLD is increasingly recognized as the hepatic manifestation of metabolic syndrome, these data reinforce the plausibility of a causal relationship. These findings are consistent with the 2025 Italian Society of Human Nutrition (SINU) food pyramid, which recommends moderate coffee consumption (3–5 cups/day) within a healthy diet (59). However, a cautious approach is advisable for more sensitive groups such as children, adolescents, pregnant or breastfeeding women, older adults, and individuals affected by anxiety or sleep disorders. The European Food Safety Authority (EFSA) states that healthy adults should not exceed a total daily caffeine intake of 400 mg from all sources (60). A typical 30 ml espresso contains on average about 40 mg of caffeine; a moderate consumption of up to a maximum of four to six cups per day generally allows healthy adults to remain within the EFSA recommended limits. However, when using a moka pot to prepare coffee, the caffeine extraction can vary due to factors such as temperature, extraction time, and the coffee-to-water ratio. This variability makes it challenging to accurately determine the caffeine content per cup, which can range from 38.4 to 162 mg for a 30 ml serving (41).

### 4.1 Strengths and limitations

This study has notable strengths, particularly its large sample size of 1,079 participants from southern Italy, yielding findings that are representative of the general population and clinical practice. It uniquely investigated the effects of Italian-style coffee on MASLD, incorporating extensive adjustments to isolate the true impact of coffee while reducing confounding factors. However, as an observational study, it identifies associations between coffee intake and MASLD, but it does not establish causality. Additionally, self-reported dietary data may be inaccurate, despite being reviewed by trained nutritionists. Another limitation is the lack of physical activity data, which can significantly influence MASLD outcomes; however, appropriate measures such as the International Physical Activity Questionnaire (IPAQ) were deemed unsuitable due to age restrictions. These strengths and limitations should be considered, emphasizing the need for further research, especially randomized trials, to better establish causality.

## 5 Conclusion

This study found an inverse, dose-dependent association between Italian-style coffee consumption and MASLD risk, with benefits starting at 1 cup per day and peaking at a moderate intake of 4–6 cups daily. These effects likely arise from the synergistic actions of multiple bioactive compounds, influenced by the specific preparation methods characteristic of Italian coffee culture. The findings align with the recommendations of the 2025 Italian Society of Human Nutrition food pyramid, which recommends discretionary consumption of up to 3–5 cups of coffee per day. Given its cultural relevance and favorable metabolic profile, Italian-style coffee may be considered a functional food for MASLD prevention, with caution advised for sensitive populations such as children, adolescents, pregnant women, older adults, and individuals with anxiety or sleep disorders. Additional longitudinal and interventional studies are warranted to confirm causality and elucidate the underlying mechanisms.

## Data Availability

The datasets generated for this study can be found in the Figshare repository, doi: 10.6084/m9.figshare.31149076.
