# Supplementary material for: Italian style coffee consumption and metabolically dysfunctional-associated steatotic liver disease (MASLD): a cohort population study in Southern Italy
Source: Front Nutr. 2026 Mar 19;13:1797230. doi: 10.3389/fnut.2026.1797230 (PMC13044158; doi:10.3389/fnut.2026.1797230)
Supplement: Supplementary file 1 [file Table_1.docx]

Supplementary Material

**Table S1.** Demographic and lifestyle characteristics of Participants by MASLD. Nutrihep Study, Putignano (BA), Italy, 2015-2018.

| Variables | Whole Sample ^b^ | MASLD | | *p-value* ^c^ |
| --- | --- | --- | --- | --- |
|  |  | No | Yes |  |
| N (%) | 1,079 (100) | 541 (50.14) | 538 (49.86) |  |
| rMED^a^ | 8.00 (6.00-10.00) | 8.00 (6.00-10.00) | 8.00 (6.00-10.00) | 0.035 |
| Super Alcoholic Beverages (ml/day)^a^ | 0.70 (0.00-2.70) | 0.70 (0.00-2.70) | 0.70 (0.00-2.70) | 0.033 |
| Kilocalories (day)^a^ | 1,985.85  (1,606.06-2,484.59) | 2,007.6  (1,654.62-2,510.08) | 1,962.84  (1,551.76-2,432.92) | 0.028 |
| Sugar in coffee (%) |  |  |  |  |
| No | 291 (26.97) | 141 (48.45) | 150 (51.55) | 0.50 |
| Yes | 788 (73.03) | 400 (50.76) | 388 (49.24) |  |
| Milk and coffee (%) |  |  |  |  |
| No | 419 (38.83) | 208 (49.64) | 211 (50.46) | 0.79 |
| Yes | 660 (61.17) | 333 (50.45) | 327 (49.55) |  |
| Smoker (%) |  |  |  |  |
| Never/Former | 929 (86.10) | 465 (50.05) | 464 (49.95) | 0.89 |
| Current | 150 (13.90) | 76 (50.67) | 74 (49.33) |  |
| Marital Status (%) |  |  |  |  |
| Single | 142 (13.16) | 92 (64.79) | 50 (35.21) | <0.001 |
| Married or Living together | 865 (80.17) | 419 (48.44) | 446 (51.56) |  |
| Separated or Divorced | 26 ( 2.41) | 19 (73.08) | 7 (26.92) |  |
| Widower | 46 ( 4.26) | 11 (23.91) | 35 (76.09) |  |
| Education (%) |  |  |  |  |
| Primary school | 236 (21.87) | 59 (25.00) | 177 (75.00) | <0.001 |
| Secondary school | 333 (30.86) | 148 (44.44) | 185 (55.56) |  |
| High School | 374 (34.66) | 241 (64.44) | 133 (35.56) |  |
| Graduated | 136 (12.60) | 93 (68.38) | 43 (31.62) |  |
| Job occupation (%) |  |  |  |  |
| Managers & Professionals | 89 ( 8.25) | 48 (53.93) | 41 (46.07) | <0.001 |
| Craft, Agricultural and Sales Workers | 390 (36.14) | 235 (60.26) | 155 (39.74) |  |
| Elementary Occupations | 162 (15.01) | 79 (48.77) | 83 (51.23) |  |
| Housewife | 109 (10.10) | 55 (50.46) | 54 (49.54) |  |
| Pensioneers | 270 (25.02) | 87 (32.22) | 183 (67.78) |  |
| Jobless | 59 ( 5.47) | 37 (62.71) | 22 (37.29) |  |
| Family income assessment (%) |  |  |  |  |
| Totally insufficient | 22 ( 2.04) | 8 (36.36) | 14 (63.64) | 0.010 |
| Just sufficient | 141 (13.07) | 63 (44.68) | 78 (55.32) |  |
| Sufficient | 846 (78.41) | 422 (49.88) | 424 (50.12) |  |
| More than sufficient | 54 ( 5.00) | 38 (70.37) | 16 (29.63) |  |
| Good | 16 ( 1.48) | 10 (62.50) | 6 (37.50) |  |

^a^As median and Interquartile Range. ^b^Percentages calculated for the column. Otherwise, percentages are calculated for the row. ^c^Wilcoxon rank-sum tests for continuous variables to compare two groups, and the χ2 test for categorical variables. MASLD: Metabolic dysfunction-associated steatotic liver disease.
