# Supplementary material for: Italian style coffee consumption and metabolically dysfunctional-associated steatotic liver disease (MASLD): a cohort population study in Southern Italy
Source: Front Nutr. 2026 Mar 19;13:1797230. doi: 10.3389/fnut.2026.1797230 (PMC13044158; doi:10.3389/fnut.2026.1797230)
Supplement: Supplementary file 2 [file Table_2.docx]

Supplementary Material

**Table S2.** Characteristics of Participants by Daily Coffee Consumption, Nutrihep Study, Putignano (BA), Italy, 2015-2018.

| Variables | Daily Coffee Consumption | | *p-value*^b^ |
| --- | --- | --- | --- |
|  | No | Yes |  |
| N (%) | 218 (16.81) | 1,079 (83.19) |  |
| MASLD (%) |  |  |  |
| No | 127 (58.3) | 541 (50.1) | 0.029 |
| Yes | 91 (41.7) | 538 (49.9) |  |
| Gender (%) |  |  |  |
| Female | 153 (70.2) | 591 (54.8) | <0.001 |
| Male | 65 (29.8) | 488 (45.2) |  |
| Age (yrs)^a^ | 53.31 (40.16-65.09) | 55.23 (43.01-66.18) | 0.24 |
| rMED^a^ | 8.00 (6.00-10.00) | 8.00 (6.00-10.00) | 0.17 |
| Super Alcoholic Beverages (ml/day)^a^ | 0.87 (2.80) | 3.64 (11.67) | <0.001 |
| Kilocalories (day)^a^ | 1,799.28 (1,439.51-2,196.91) | 1,988.92 (1,608.65-2,484.59) | <0.001 |
| Smoker (%) |  |  |  |
| Never/Former | 209 (95.9) | 929 (86.1) | <0.001 |
| Current | 9 ( 4.1) | 150 (13.9) |  |
| Marital Status (%) |  |  |  |
| Single | 39 (17.9) | 142 (13.2) | 0.17 |
| Married or Living together | 169 (77.5) | 865 (80.2) |  |
| Separated or Divorced | 2 ( 0.9) | 26 ( 2.4) |  |
| Widower | 8 ( 3.7) | 46 ( 4.3) |  |
| Education (%) |  |  |  |
| Primary school | 46 (21.1) | 236 (21.9) | 0.067 |
| Secondary school | 50 (22.9) | 333 (30.9) |  |
| High School | 86 (39.4) | 374 (34.7) |  |
| Graduated | 36 (16.5) | 136 (12.6) |  |
| Job occupation (%) |  |  |  |
| Managers & Professionals | 13 ( 6.0) | 89 ( 8.2) | 0.14 |
| Craft, Agricultural and Sales Workers | 79 (36.2) | 390 (36.1) |  |
| Elementary Occupations | 23 (10.6) | 162 (15.0) |  |
| Housewife | 32 (14.7) | 109 (10.1) |  |
| Pensioneers | 55 (25.2) | 270 (25.0) |  |
| Jobless | 16 ( 7.3) | 59 ( 5.5) |  |
| Family income assessment (%) |  |  |  |
| Totally insufficient | 5 ( 2.3) | 22 ( 2.0) | 0.98 |
| Just sufficient | 26 (11.9) | 141 (13.1) |  |
| Sufficient | 173 (79.4) | 846 (78.4) |  |
| More than sufficient | 10 ( 4.6) | 54 ( 5.0) |  |
| Good | 4 ( 1.8) | 16 ( 1.5) |  |

^a^As median and Interquartile Range. ^b^Wilcoxon rank-sum tests for continuous variables to compare two groups, and the χ2 test for categorical variables. MASLD: Metabolic dysfunction-associated steatotic liver disease; rMED: Relative Mediterranean Diet; BMI: Body Mass Index; SBP: Systolic Blood Pressure; DBP: Diastolic Blood Pressure; HbA1c: Glycosylated Haemoglobin; HOMA: Homeostasis Model Assessment; ALT: Alanine Amino transferase; ɣGT: Gamma Glutamyl Transferase; AST: Aspartate Amino transferase; TG: Triglycerides; TC: Total Cholesterol; HDL-C: High-Density Lipoprotein Cholesterol; ALP: Alkaline Phosphatase Level.
