# Supplementary material for: Italian style coffee consumption and metabolically dysfunctional-associated steatotic liver disease (MASLD): a cohort population study in Southern Italy
Source: Front Nutr. 2026 Mar 19;13:1797230. doi: 10.3389/fnut.2026.1797230 (PMC13044158; doi:10.3389/fnut.2026.1797230)
Supplement: Supplementary file 3 [file Table_3.docx]

Supplementary Material

**Table S3.** Logistic regression analysis of the association between daily coffee consumption and MASLD, including confounding variables.

|  | OR^a^ | *p-value* | 95% CI |
| --- | --- | --- | --- |
| Daily coffee consumption: | |  |  |
| <1 cup | 1.000 |  |  |
| 1 cup | 0.479 | 0.033 | 0.244; 0.944 |
| 2 cups | 0.468 | 0.024 | 0.242; 0.905 |
| 3 cups | 0.449 | 0.023 | 0.225; 0.893 |
| 4-6 cups | 0.407 | 0.025 | 0.186; 0.893 |
| Sex | 1.052 | 0.790 | 0.723; 1.530 |
| BMI | 1.286 | <0.001 | 1.229; 1.346 |
| Age | 1.058 | <0.001 | 1.042; 1.074 |
| Education | 0.788 | 0.016 | 0.650; 0.956 |
| Glycemic categories | 1.368 | 0.064 | 0.982; 1.906 |
| AST/ALT | 0.339 | <0.001 | 0.180; 0.639 |
| Daily Energy | 1.000 | 0.421 | 1.000; 1.000 |
| Sugar in coffee | 0.860 | 0.416 | 0.598; 1.236 |
| Smoke | 1.571 | 0.064 | 0.973; 2.537 |
| rMED | 0.906 | 0.439 | 0.704; 1.164 |
| Superalcoholic | 0.996 | 0.653 | 0.979; 1.013 |
| DBP | 1.018 | 0.105 | 0.996; 1.040 |
| HDL-C | 0.989 | 0.124 | 0.975; 1.003 |
| Milk and coffee | 0.777 | 0.147 | 0.553; 1.092 |

^a^No MASLD reference category. The data are reported as Odds Ratios (ORs) and 95% C.I. Model adjusting for age, sex (Female vs. Male), Weight, Diastolic Blood Pressure, Daily Energy, Sugar in coffee (No vs Yes), Milk and coffee (No vs Yes); Education, Smoke (never vs. current), rMED, Superalcoholic (ml/day), HDL-C, AST/ALT, and Glycemic categories (normoglycemia <100 mg/dL, prediabetes 100–125 mg/dL, diabetes ≥126 mg/dL). **Legend**: rMED: Relative Mediterranean Diet; DBP: Diastolic Blood Pressure; HDL-C: High-Density Lipoprotein; AST: Aspartate Amino Transferase; ALT: Alanine Amino Transferase; HOMA: Homeostasis Model Assessment.
