# Supplementary figures and images for: Italian style coffee consumption and metabolically dysfunctional-associated steatotic liver disease (MASLD): a cohort population study in Southern Italy
Source: Front Nutr. 2026 Mar 19;13:1797230. doi: 10.3389/fnut.2026.1797230 (PMC13044158; doi:10.3389/fnut.2026.1797230)

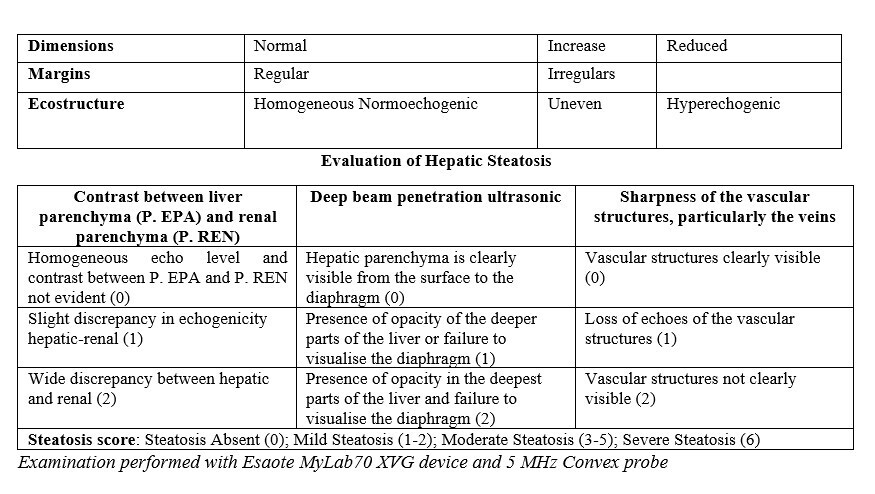

Supplement: Supplementary file 4 [file Image_1.jpeg]
